# Mechanisms of Action of the Antimicrobial Peptide Cecropin in the Killing of *Candida albicans*

**DOI:** 10.3390/life12101581

**Published:** 2022-10-11

**Authors:** Cui Peng, Yang Liu, Liangyong Shui, Zhongyi Zhao, Xinfang Mao, Zhongyuan Liu

**Affiliations:** College of Chemical Engineering, Sichuan University of Science and Engineering, 180 Xueyuan Street, Zigong 643000, China

**Keywords:** antimicrobial peptides, *Candida albicans*, antifungal activity, cell membrane, cell wall, mitochondria

## Abstract

The development of drug resistance has caused fungal infections to become a global health concern. Antimicrobial peptides (AMPs) offer a viable solution to these pathogens due to their resistance to drug resistance and their diverse mechanisms of actions, which include direct killing and immunomodulatory properties. The peptide Cecropin, which is expressed by genetically engineered bacteria, has antifungal effects on *Candida albicans*. The minimal inhibitory concentration (MIC) and the minimal fungicidal concentration (MFC) of *Candida albicans* were 0.9 μg/mL and 1.8 μg/mL, respectively, detected by the micro-broth dilution method. According to the killing kinetics, the MFC of Cecropin could kill *Candida albicans* in 40 min. The electron microscope indicated that Cecropin could cause the cell wall to become rough and nicked, eventually killing *Candida albicans*. The effects of Cecropin on the cell membrane of treated *C. albicans*, using the 1,6-diphenyl-1,3,5-hexatriene and propidium iodide protocol, showed that they could change the permeability and fluidity, destroy it, and lead to cell necrosis. In addition, Cecropin can also induce cells to produce excessive reactive oxygen species, causing changes in the mitochondrial membrane potential. Therefore, this study provides a certain theoretical basis for the antifungal infection of new antifungal agents.

## 1. Introduction

Antimicrobial peptides (AMPs) are small proteins with biological activity, and they play an important role in the body’s immunity as the first line of defense [1]. They not only have a strong killing effect on bacteria, but also have killing activity on fungi, viruses and tumors [2,3,4,5]. The antibacterial effects of AMPs mainly include the direct killing and immunomodulatory properties. The initial interaction with the cell membrane is imperative for the direct antimicrobial property by AMPs, since the amphiphilic structure of antimicrobial peptides can bind to and destroy the cell membrane, resulting in changes in the permeability of the cell membrane and even the outflow of intracellular macromolecular substances [6,7]. In addition, antifungal peptides induce cell wall fragility by binding to chitin, which forms the fungal cell wall. [8]. Moreover, some AMPs have been found to penetrate into cells and interact with fungal mitochondria and nucleic acids to cause cell death [9]. In addition, many AMPs have been shown to regulate a broad range of immunomodulatory activities, which include suppressing proinflammatory cytokines, enhancing the recruitment of leukocytes, and stimulating the secretion of neutrophil chemokines [10,11]. Overall, many AMPs likely function through multiple complementary actions, which may be partially responsible for AMPs not only having antibacterial activity against drug-resistant strains but also not being prone to drug resistance [12]. Therefore, antimicrobial peptides have attracted much attention as a kind of natural small-molecule substance.

The *Candida albicans*, also known as *Candida*, is round or elliptical and one of the main pathogenic fungi [13]. *Candida albicans* widely exists in nature and is widely distributed in symbiotic state on the oral cavity, digestive tract, vagina, and skin surface of healthy people, which can often cause mucosal diseases and blood infection [14]. *Candida albicans* is usually a harmless symbiotic fungus which can become opportunistic microorganisms in immunocompromised or immunocompromised individuals. It is considered to be opportunistic infection fungus. In the United States, *Candida* is the fourth largest pathogen of hospital-acquired bloodstream infections, and about 50% of infection cases are caused by *Candida albicans* [15]. As a commensal pathogen, *Candida* can quickly adapt to environmental changes in the host even when nutrient bioavailability is limited [16]. In recent years, the number of fungal infections has increased significantly, and such infections usually occur in patients with low immunity or severe illness; with the abuse of antibiotics, the development of drug resistance has become the current challenge in the treatment of fungal infections [17]. At present, the commonly used drugs for the treatment of fungal infection mainly include azoles, echinocandins, and polyenes, but their treatment brings certain toxic side effects [18]. For example, fluconazole, the most commonly used antifungal drug in clinics, has developed severe drug resistance due to long-term large-scale use; the long-term use of amphotericin B, nystatin, and ketoconazole will cause side effects such as hepatotoxicity and nephrotoxicity; the echinocandin drugs are expensive and have severe allergic reactions; and 5-fluorocytosine has severe adverse reactions such as bone-marrow suppression and liver-function damage [17,19]. Efforts to develop alternative antifungal agents have therefore become necessary.

At present, many antibacterial peptides have been found to have good antifungal activity, and their antibacterial mechanisms have been reported. The artificially synthesized 14-hellical B-peptides can bind to the cell membrane of *Candida albicans* through hydrophobic and electrostatic interactions and insert into and penetrate the cell membrane in a synergistic manner when reaching the critical threshold concentration; they then enter the cell and destroy organelles, leading to cell death [20]. Liu Huifan et al. found that the polypeptides Asp-Tyr-Asp-Asp (DYDD) and ASP-ASP-Tyr (DDDY) extracted from *Gynostemma pentaphyllum* exert antibacterial activity by damaging the cell membrane and causing content leakage [21]. In addition, Melittin can induce apoptosis of *Candida albicans* due to the increase of reactive oxygen species, phosphatidylserine externalization, DNA, and nuclear fragmentation [22].

Therefore, in order to understand the antifungal mechanism of Cecropin against *Candida albicans*, we expressed Cecropin by using a genetically engineered strain preserved in the laboratory. First, the MIC and MFC of *Candida albicans* treated with the peptide was measured, and the fungicidal kinetic curve was measured. The changes of cell morphology and cell wall were detected by scanning electron microscopy and transmission electron microscopy. The damage to the cell membrane of *Candida albicans* was analyzed by using a multifunctional microplate reader and fluorescence microscope. Finally, the accumulation of ROS and the change of mitochondrial membrane potential were detected by fluorescent probes, which provided a certain experimental basis for its antifungal infection.

## 2. Results

### 2.1. Antifungal Activity

In order to investigate the activity against *Candida albicans,* the MIC and MFC of Cecropin were determined. The MIC and MFC of Cecropin against *Candida albicans* were 0.9 μg/mL and 1.8 μg/mL, respectively, and the MIC and MFC values of the control, amphotericin B (AMB), were 1.875 μg/mL and 3.75 μg/mL, respectively.

### 2.2. Time–Killing Assay

**Figure 1:**
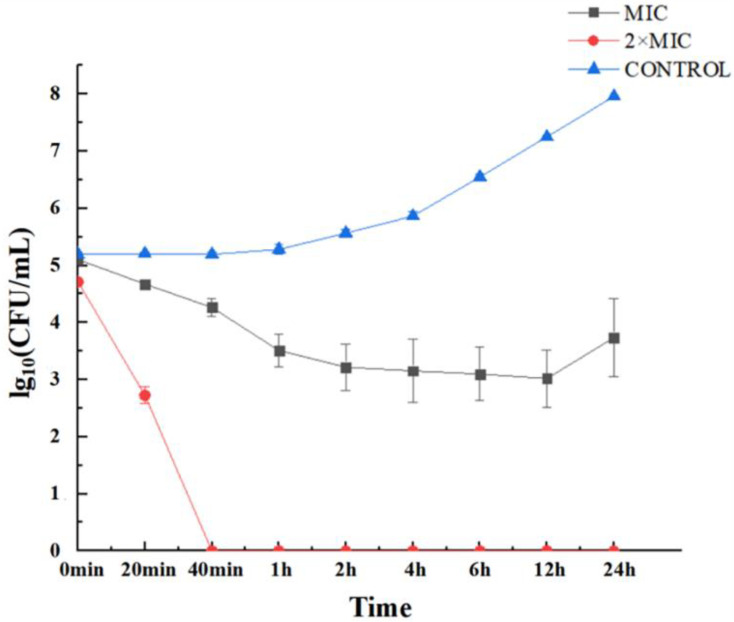
Time–kill kinetics of MIC and 2 × MIC Cecropin in *Candida albicans*. Experiments were conducted in YPD medium with an incubation period of 24 h at 28 °C. Samples taken at specified times were evaluated for colony-forming units (CFUs). These values were the mean of the independent triplicate replicates, showing an error bar (*p* < 0.05).

Time–killing kinetics is an important parameter to judge the performance of antimicrobial peptides. The results were as shown in the Figure 1. After treatment with concentration of 2 × MIC, the number of viable bacteria was dramatically decreased, and *Candida albicans* could be completely killed within 40 min. The killing kinetics curve of Cecropin treated with MIC concentration firstly decreased and then increased, and the results showed that Cecropin with MIC concentration could not completely kill *Candida albicans*, even after incubation for 24 h.

### 2.3. Scanning-Electron-Microscope Studies

**Figure 2:**
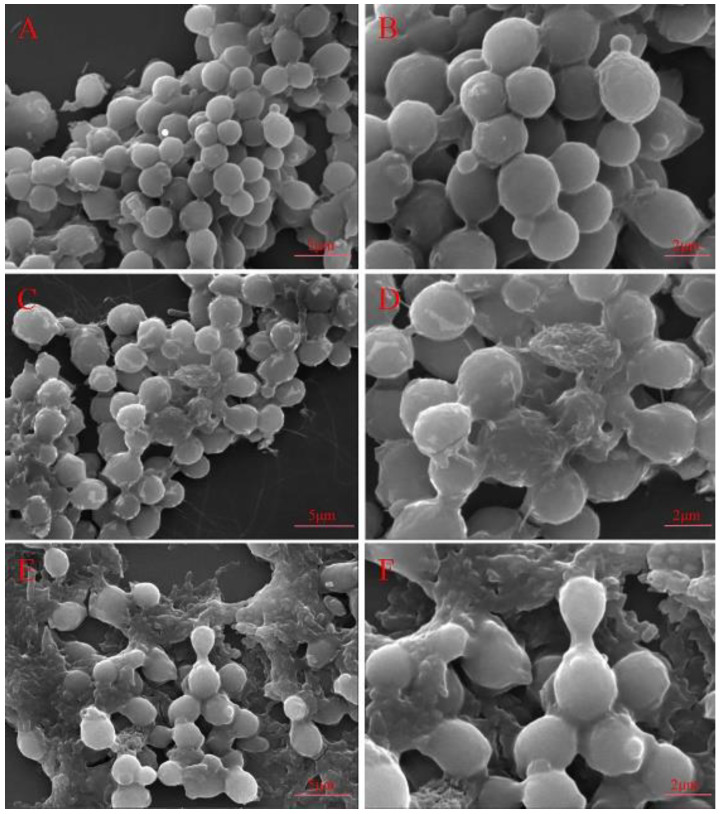
Scanning electron micrographs of *Candida albicans* either untreated or treated with Cecropin: (**A**,**B**) untreated control, (**C**,**D**) treatment with 1.8 μg/mL Cecropin for 30 min, and (**E**,**F**) treatment with 1.8 μg/mL Cecropin for 60 min.

In order to further characterize the role of Cecropin, the effect of treatment with Cecropin on the cell morphology of *Candida albicans* was observed under a scanning electron microscope. The results are as shown in Figure 2. The cells were treated with 1.8 μg/mL peptide, and the results showed that, after 30 min, the cell-surface roughness was increased, and the cells began to shrink (Figure 2C,D). After 60 min of treatment, the cell structure was changed significantly, and a large number of cells were aggregated and dissolved (Figure 2E,F). However, the cells in the control group were complete in morphology, and the surface was smooth, round, or elliptical, with no dissolution (Figure 2A,B).

### 2.4. Transmission Electron Microscopy

**Figure 3:**
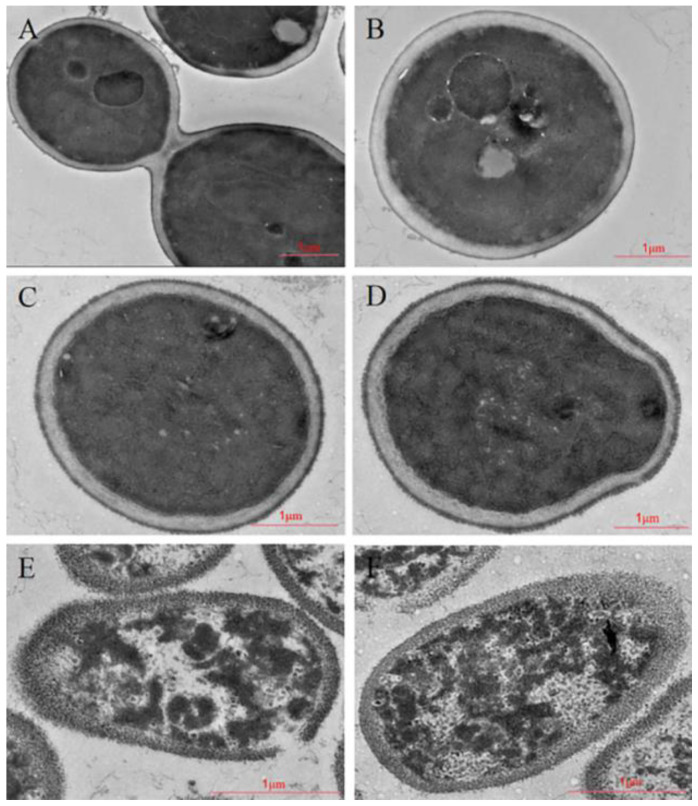
Transmission electron micrographs showing *Candida albicans* (ATCC-90028) cell wall damage by Cecropin: (**A**,**B**) untreated control, (**C**,**D**) treatment with 1.8 μg/mL Cecropin for 40 min, and (**E**,**F**) treatment with 1.8μg/mL Cecropin for 60 min.

Through transmission-electron-microscope analysis, the ultrastructure of *Candida albicans* was changed obviously under the action of Cecropin, as shown in Figure 3. The cell wall untreated with Cecropin was smooth, and the structures of the cell wall and cell membrane were intact. The cell wall and cell membrane were translucent, and the cytoplasm was uniform and full. After treatment with the peptides Cecropin at 1.8 μg/mL for 30 min, the surface of the cell wall became significantly rough, there was no transparency between the cell wall and cell membrane, and there were white spots in the cells (Figure 3C,D). After 60 min of treatment, the gap between the cell wall and cell membrane disappeared, the cell wall gradually dissolved, an obvious gap appeared, the cytoplasm in cells was uneven, the cytoplasmic structure was disordered, and severe cell necrosis could be observed. The results showed that Cecropin could destroy the morphology and structure of *Candida albicans* cells, and the damage became more and more serious with time (Figure 3E,F).

### 2.5. Determination of Alkaline Phosphatase

**Figure 4:**
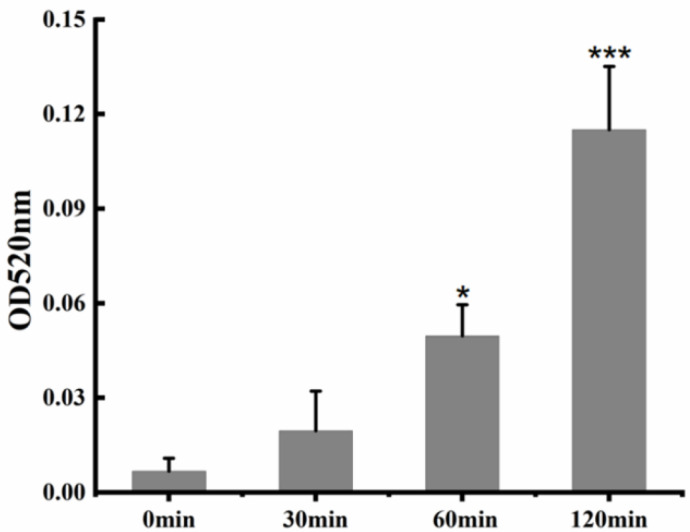
Effects of Cecropin on AKP enzyme activity of *Candida albicans*. These values were the means of the independent triplicate replicates, showing an error bar. The Tukey test was used for the analysis-of-variance comparison; * = *p* < 0.05 and *** = *p* < 0.001.

AKP is a phosphatase that is present between the cell wall and the cell membrane, and it can be used as an indicator of cell-wall permeability. As shown in Figure 4, with the increase of incubation time of Cecropin and *Candida albicans*, the activity of extracellular AKP enzyme also gradually increased. The one-way analysis of variance showed that after incubation of Cecropin with *Candida albicans* for 60 min and 120 min, compared with the control group, the significant increase of extracellular AKP activity was 7.3-fold and 16.9-fold, respectively. The above data indicated that the cell wall was damaged or even destroyed by the polypeptide treatment of Rhizoma Gastrodiae, resulting in the leakage of the AKP enzyme, which was consistent with the observation results under the electron microscope.

### 2.6. Membrane Permeability of Candida albicans

**Figure 5:**
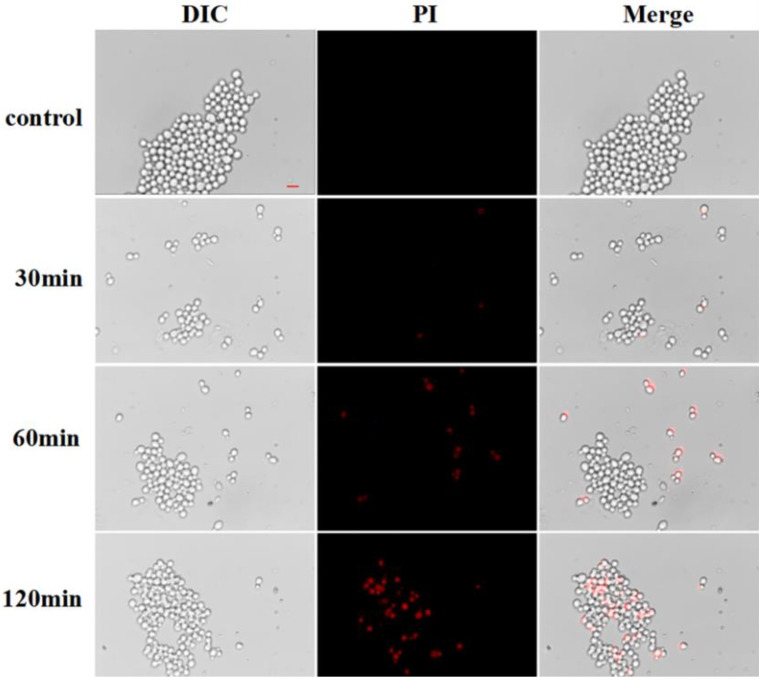
Inverted fluorescence microscope analysis of membrane permeabilization assay by PI uptake. Control indicated that the samples were not treated with Cecropin, and the treatment time was 0 min. The scale bar represents 5 μm.

Propidium iodide (PI) was a fluorescent dye. It could not enter the cells with intact cell membrane, but it could enter the cells with damaged membrane structure and emit red fluorescence. Based on this characteristic, a fluorescence microscope observation was conducted. When the permeability of the cell membrane is increased, more PI dye will enter the cell, thus reflecting the integrity of the cell membrane. The observation showed that no PI fluorescence was observed in the *Candida albicans* control group without peptide treatment, indicating that the cell membrane of *Candida albicans* was intact. In the presence of 1.8 μg/mL of Cecropin, the absorption of PI by *Candida albicans* gradually increased with the increase of incubation time. When the time increased to 120 min, the red fluorescent cells of *Candida albicans* were the most (Figure 5). These results indicated that the cell membrane of *Candida albicans* was damaged and that permeability was increased after the treatment with Cecropin.

### 2.7. Detection of Cell-Membrane Fluidity

**Figure 6:**
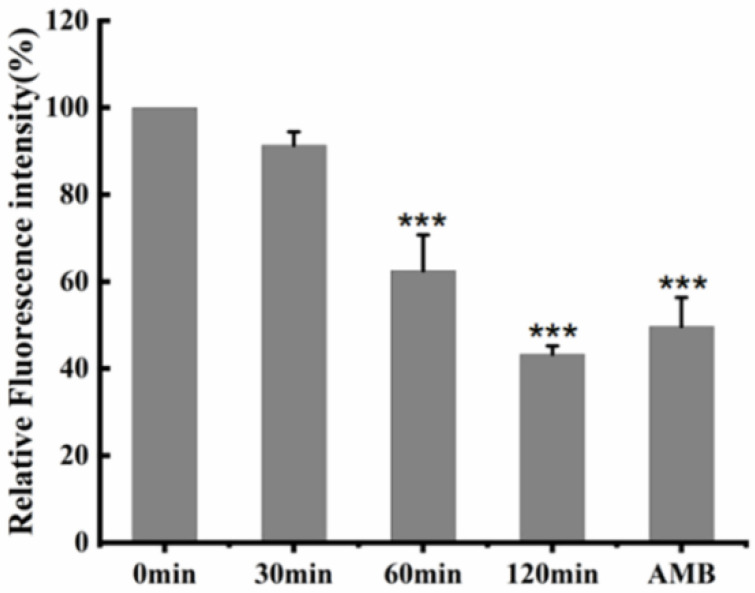
DPH fluorescence intensity after peptide treatment on *Candida albicans*. Subcultured cells containing Cecropin (1.8 μg/mL) were incubated at 28 °C for 0 min, 30 min, 60 min, and 120 min. These values were the means of the independent triplicate replicates, showing an error bar; *** = *p* < 0.001.

To further investigate the active mechanism of Cecropin, we used the fluorescent probes 1,6-diphenyl-1,3,5-hexatriene (DPH) to obtain information about its membrane fluidity. DPH is a hydrophobic molecule that allows it to bind to the hydrocarbon tail region of phospholipids in the cytoplasmic membrane without interfering with the structure of the lipid bilayer. Such fluorescent probes are commonly used not only to study the structure of membranes, but also to study the biophysical properties of membranes. In addition, the fluidity of the film can also be measured by fluorescence spectral analysis, using DPH molecules. If the anti-*Candida albicans* activity of the antimicrobial peptide causes membrane damage by membrane depolarization, the DPH molecule cannot bind to the lipophilic tail of the phospholipid in the lipid bilayer. As shown in Figure 6, with the increase of incubation time between the peptide and *Candida albicans*, the fluorescence intensity of DPH gradually decreased, and the one-way analysis of variance found that, compared with the control, the incubation time of 60 min and 120 min had significant differences, which decreased by 37.45% and 56.65%, respectively. The positive control, amphotericin B, decreased by 50.28%, indicating that Cecropin could significantly change the cell-membrane fluidity of *Candida albicans* after 60 min of treatment.

### 2.8. Reactive Oxygen Species

**Figure 7:**
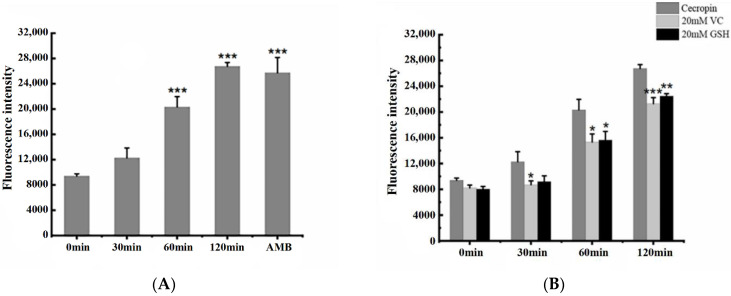
*Candida albicans* treated with 2 × MIC Cecropin produced ROS levels at different time points. (**A**) Efficacy of Cecropin on ROS of *Candida albicans* cells, as shown by multifunctional fluorescent microplate reader analysis. (**B**) Effect of antioxidants VC and GSH on Cecropin-induced ROS generation. These values were the means of the independent triplicate replicates, showing an error bar. The Tukey test was used for the analysis-of-variance comparison; * = *p* < 0.05, ** = *p* < 0.01, and *** = *p* < 0.001.

The formation of reactive oxygen species (ROS) is considered to be one of the bactericidal mechanisms of many antibacterial peptides. Dye DCFH-DA can freely pass through the cell membrane and enter the cells to be oxidized into DCFH, which cannot pass through the cell membrane, thus allowing the probe to be easily loaded into the cells. Cellular reactive oxygen species can oxidize non-fluorescent DCFH to generate DCF, producing green fluorescence, and the level of intracellular reactive oxygen species can be known by detecting the fluorescence of DCF. After *Candida albicans* was incubated with Cecropin at a concentration of 2 × MIC for different times, ROSs produced by Cecropin were detected, as shown in Figure 7A. Compared with the untreated control, the ROS levels gradually increased with the passage of time. Through the one-way analysis of variance, it was found that the treatment of *Candida albicans* with Cecropin for 60 min and 120 min had a significant difference compared with the control (0 min) ratio and increased by 2.16 and 2.85 times, respectively. The positive control, amphotericin B, also increased 2.74 times, indicating that Cecropin can induce the production of reactive oxygen species in *Candida albicans*. In the presence of the antioxidant VC, the ROS levels were significantly decreased at 30 min, 60 min, and 120 min compared with the control group, while in the presence of GSH antioxidants, the ROS levels were significantly decreased at 60 min and 120 min, indicating that the addition of VC and GSH could inhibit the production of ROSs induced by Cecropin (Figure 7B).

### 2.9. Mitochondrial Membrane Potential Detection

**Figure 8:**
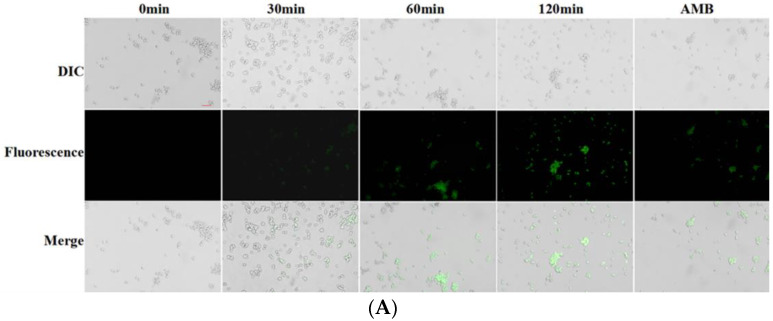

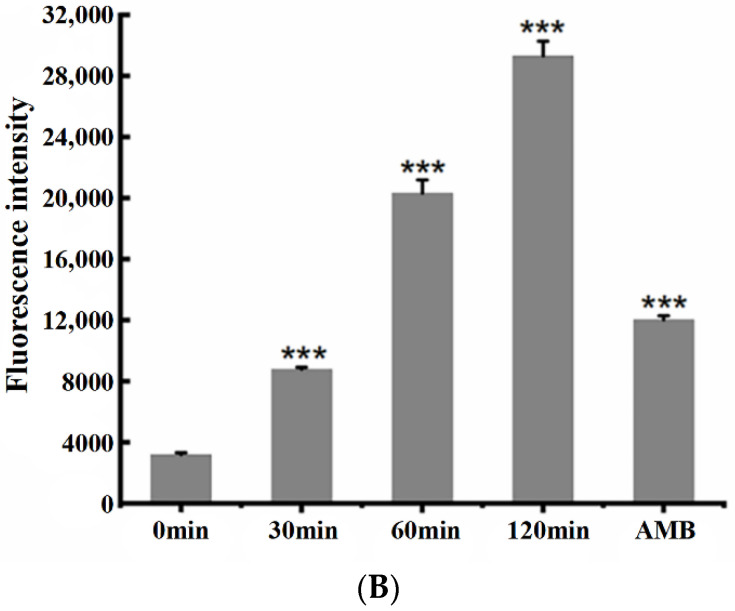
The effect of Cecropin on mitochondrial membrane potential of *Candida albicans*. (**A**) Fluorescence-microscope observation of *Candida albicans* after DCFH-DA staining with different treatments. The scale bar represents 10 μm. (**B**) Fluorescence intensity was measured to indicate the mitochondrial membrane potential by multifunctional microplate reader. These values were the means of the independent triplicate replicates, showing an error bar. The Tukey test was used for the analysis-of-variance comparison; *** = *p* < 0.001.

Mitochondrial membrane potential, as an indicator of the cell’s energy state, can reflect the proton loading in mitochondria, the activity of electron transporters, and the permeability of mitochondrial membrane. We used rhodamine as a probe to detect the effect of Cecropin on the mitochondrial-membrane potential of *Candida albicans* strains. The results of the multifunctional microplate reader showed that the fluorescence intensities of Cecropin treated for 30 min, 60 min, and 120 min were 2.75-times, 6.35-times, and 9.16-times higher than those of the untreated ones, respectively, while the fluorescence intensity of the positive control, amphotericin B, increased only 3.76 times after 2 h treatment. The results of the inverted fluorescence microscope were consistent with those of Figure 8B, where the fluorescence intensity was significantly enhanced at a concentration of 1.8 μg/mL of the peptide Cecropin (Figure 8A). These results indicated that Cecropin could induce the hyperpolarization of the mitochondrial membrane potential and induce mitochondrial damage.

## 3. Discussion

Antimicrobial peptides are a class of small-molecule polypeptides produced by organisms, and they are important components of the natural immune defense system [1,23]. In a previous study, the peptide Cecropin was found to be active against *Candida albicans*, so its antifungal activity was tested, and it was found that the peptide Cecropin was completely killed in a short time, at a concentration of 1.8 μg/mL, with a rapid effect. In view of the bactericidal activity of Cecropin and its almost-nonexistent drug resistance, research on its action mechanism was continued.

In order to further observe the antifungal effect of Cecropin, we first observed the changes of *Candida albicans* under a scanning electron microscope and a transmission electron microscope. The results of the scanning electron microscopy showed that the cell surface became rough and wrinkled, and dissolution occurred with time. In addition, the results of the transmission electron microscopy revealed that the cell wall, cell membrane, and intracellular organelles might be the target of action. The cell wall is the first protective barrier of fungi, serving as a physical and chemical barrier to protect cells, and it is crucial to maintaining cell integrity and viability [24]. Maurya et al. found that the synthetic cationic peptides VS2 and VS3, when applied to *Candida albicans*, could wrinkle the cell surface and break the cell wall, thus exerting the antibacterial effect [25]. In this study, the measurement of AKP enzyme showed that the enzyme activity increased gradually with time, indicating the leakage of the AKP enzyme and revealing that the cell wall was the target of Cecropin action.

Although the amino acid sequences of different antimicrobial peptides are highly heterogeneous and the secondary structures are diverse, most antimicrobial peptides are cationic and amphiphilic [26]. At present, it has been found that the activity against *Candida albicans* is mainly through its cationic and amphiphilic binding or insertion into the pathogen microorganism, where it destroys and disrupts the cytoplasmic membrane [27]. The cell membrane, as its second protective barrier, is mainly composed of a lipid bilayer, and most cationic antibacterial peptides exert an antifungal effect by acting on the cell membrane [28]. Therefore, we observed the effect of Cecropin on the fungal plasma membrane, and the fluorescent dyes DPH and PI were indicators of the cell membrane dynamics and permeability of *Candida albicans*, respectively. DPH, a hydrophobic molecule, is labeled in the lipid layer, mainly reflecting the fluidity and ordering of the cell membrane. After being treated with Cecropin for different amounts of time, the fluorescence intensity of DPH decreased significantly, indicating that Cecropin could act on the lipid layer of the cell membrane [29]. When propidium iodide (PI, a fluorescent dye that cannot cross the entire cell membrane) was incubated, the increased fluorescence indicated increased membrane permeability, revealing the damage to the cell membrane of *Candida albicans* [30].

The production of ROS contributes to the general action mechanism of antifungal drugs and many peptides on pathogenic fungi [31]. In this study, the intracellular ROS content of *Candida albicans* was determined by using the active oxygen-detection kit (DCFH-DA dye), we found that, with the increase of the treatment time of Cecropin, it could induce *Candida albicans* to produce excessive active oxygen, indicating mitochondrial dysfunction. Moreover, the addition of antioxidants VC and GSH glutathione could reduce the content of active oxygen, indicating that antioxidants could reverse the production of active oxygen. Therefore, we speculate that ROS production also leads to the antifungal activity of Cecropin. Reactive oxygen species (ROS) are byproducts of cell metabolism and mainly present in the mitochondria. A low concentration of ROS acts as a signal transduction molecule to maintain the normal activity of cells. When *Candida albicans* meets external pressure such as oxidant or heat shock, it will produce reactive oxygen to cope with the pressure. However, when it cannot eliminate the produced reactive oxygen, excessive reactive oxygen will cause cytotoxicity, leading to damage to DNA, protein, lipids, and other biological macromolecules, and leading to apoptosis or necrosis [32,33]. Delattin N et al. confirmed that various antifungal drugs caused oxidative damage to *Candida albicans* [34]. Pleurocidin is an antibacterial peptide that can induce the apoptosis of *Candida albicans* through oxidative stress [35].

In this study, rhodamine 123 was used to detect the changes in mitochondrial membrane potential of *Candida albicans*, and the fluorescence intensity was significantly increased compared with that of the control, indicating that Cecropin could hyperpolarize mitochondrial membrane potential. The mitochondrion, the central site of production capacity in eukaryotic cells, plays an important role in maintaining cell energy metabolism and regulating cell growth, differentiation, and death. Therefore, the change of membrane potential is also one of the mechanisms of antifungal activity. The production of ROS is also in the mitochondria. Excessive ROS will lead to lipid peroxidation of the mitochondrial membrane, changing the membrane performance and, eventually, the membrane potential [36]. Therefore, the increase of reactive oxygen species and the change of membrane potential are the causes of mitochondrial damage. In short, the multiple action patterns of Cecropin suggest that it can become a potential therapeutic solution for infection caused by fungal strains. This study may provide a theoretical basis for the development of new antibiotics.

## 4. Materials and Methods

### 4.1. Peptides

In this study, the peptide Cecropin was expressed in YPD medium by using genetic-engineering bacteria preserved in the laboratory under the expression condition of 30 °C, 180 rpm for 24 h [37], and the separation and purification were carried out by three-step chromatography with reference to the method of Dou et al. [38] and identified by high-performance liquid chromatography [39].

### 4.2. Fungal Strains and Growth Media

*Candida albicans* strain CMCC(F)98001 (Purchased from Ningbo Mingzhou Biotechnology Co., Ltd., Ningbo, China) was used for all experiments. The strain was maintained on a yeast-peptone-dextrose (YPD; 10 g/L yeast extract, 20 g/L peptone, 20 g/L dextrose) agar plate. Prior to use in experiments, single colony from *C. albicans* was used to inoculate YPD liquid medium, and the culture was grown overnight at 28 °C in a shaker at 180 rpm.

### 4.3. Antifungal Activity Assay

#### 4.3.1. Determination of Minimal Inhibitory Concentration (MIC)

In this study, minimal inhibitory concentration was determined by the broth microdilution method [40]. *Candida albicans* (CMCC(F)98001) (1 × 10^5^–5 × 10^5^ CFU/mL) were inoculated in YPD medium; the peptides were also diluted in a twofold gradient to 3.6–0.1125 μg/mL, *Candida albicans* and the diluted Cecropin were added in a ratio of 1:1. The 96-well plate was incubated at 28 °C for 48 h, and absorbance was assessed at 600 nm by using a microplate reader. The minimum inhibitory concentration was defined as the lowest concentration that could 100% inhibit the growth of fungi. The experiment was conducted in triplicate.

#### 4.3.2. Determination of Minimum Fungicidal Concentration (MFC)

Using a 96-well plate for MIC determination, the solutions (50 μL) in the 1 × MIC and 2 × MIC concentration wells of the plate were added to a YPD agar plate for culture for 48 h, and the colonies were counted for the test of three parallel samples. The MFC was the lowest concentration that showed either no growth or fewer than three colonies to obtain approximately 99 to 99.5% killing activity.

### 4.4. Time–Killing Assay

*Candida albicans* (CMCC(F)98001) (1–5 × 10^6^ CFU/mL) were inoculated in YPD medium, and then 0.9 μg/mL and 1.8 μg/mL Cecropin were prepared. Cecropin and the bacterial solution were mixed and cultured in a volume of 1:1. Then the time points of 0 min, 20 min, 40 min, 1 h, 2 h, 4 h, 6 h, 12 h, and 24 h were selected, and suitable dilutions were plated on YPD agar plate for culture at 28 °C for 24 h. The number of colonies was counted, and the sterilization kinetics curve was drawn [41]. Non-antimicrobial peptide was treated as the control. The experiment was conducted in triplicate.

### 4.5. Damage of Cecropin to Cell Wall of Candida albicans

#### 4.5.1. Scanning Electron Microscope

*Candida albicans* (CMCC(F)98001) (1–5 × 10^6^ CFU/mL) were inoculated in YPD medium, and the equal volume of polypeptide and bacterial solution with the concentration of 1.8 μg/mL was incubated in a constant temperature incubator at 28 °C for 30 min and 1 h, without treatment with Cecropin served as the control group. The cells were taken out, centrifuged at 5000 r/min for 5 min, washed with PBS buffer for three times, and the supernatant was discarded. The precipitate was added with an equal volume of 2.5% glutaraldehyde, blown evenly, and fixed at 4 °C overnight. After centrifugation at 5000 rpm for 10 min, the supernatant was discarded and resuspended in PBS buffer three times. The samples were dehydrated with aqueous ethanol solution at the concentration gradient of 30%, 50%, 70%, 80%, and 90%. After the aqueous ethanol solution was orderly added, the samples were allowed to stand for 10 min, centrifuged at 3000 rpm for 10 min, and finally dehydrated with pure ethanol. After the treated samples were dried and sprayed with gold, they were observed under a scanning electron microscope [42].

#### 4.5.2. Transmission Electron Microscope

In an ultrastructural study, the *Candida albicans* sample to be tested was obtained by following the same method as the scanning electron microscope. The sample was fixed overnight with 2.5% glutaraldehyde prepared with 0.1 M phosphate buffer, washed three times with PBS buffer after fixation, and fixed at 4 °C for 2 h with 1% erucic acid; it was then washed three times with PBS again. The samples were successively dehydrated in a gradient of 30%→50%→70%→90%→100% with ethanol, Epon 812 immersed, embedded, and polymerized. The ultrathin sections were prepared by an ultrathin microtome and double stained with uranium acetate and lead citrate. The ultrastructural changes were observed by a transmission electron microscope [42].

#### 4.5.3. Determination of Alkaline Phosphatase Activity

After incubating a mixture containing Cecropin (final concentration: 1.8 μg/mL) (treatment group) or an equal volume of PBS (control group) in fungal suspension (1–5 × 10^6^ CFU/mL) for 30 min, 60 min, and 120 min, samples for the detection of extracellular alkaline phosphatase (AKP) activity were collected from the supernatant of each strain in the reaction system. The activity was detected by the Beyotime AKP assay kit of Nanjing Jiancheng Bioengineering Institute (Nanjing, China) [43]. The experiment was conducted in triplicate.

### 4.6. Membrane Permeabilization Assay Using Fluorescent Inverted Microscope

In this study, the PI staining kit of Sangon Biotech (Shanghai, China) was used to test the membrane permeability of *Candida albicans* treated with Cecropin [44]. *Candida albicans* in the logarithmic phase was mixed with a 1.8 μg/mL concentration of Cecropin in equal volume and incubated in an incubator at 28 °C for 30 min, 60 min, and 120 min, respectively. Those that were not treated with the peptide served as the control group. After incubation, the cells were washed twice with phosphate-buffered saline, at pH = 7.2–7.4, and then re-suspended with an appropriate amount of 1 × assay buffer to adjust the cell concentration to 10^6^ CFU/mL. Then 95 μL of the mixed solution was added with 5 μL of PI dye and stained in the dark at room temperature for 25 min. After being washed twice with PBS, 15 μL of bacterial solution was taken and placed in the center of the glass slide. The cover glass was pressed into tablets and allowed to stand for 3–5 min before being photographed under a fluorescence microscope.

### 4.7. Detection of Cell Membrane Fluidity

Fluorescence intensities from the plasma membrane of fungal cells labeled with 1,6-diphenyl-1,3,5-hexatriene (DPH) were examined to investigate the activity of Cecropin on the membrane [29]. Specifically, *Candida albicans* cells (1–5 × 10^6^ CFU/mL) were incubated with Cecropin at a concentration of 2 × MIC at 28 °C and taken out at the time points of 30 min, 60 min, and 120 min, respectively. Samples of the fungal cultures were fixed by 0.37% formaldehyde, collected, and then washed with PBS. The cells were frozen by using liquid nitrogen. For labeling purposes, cells were thawed with PBS and resuspended in PBS. The suspended mixture was incubated with a final concentration of 0.6 mM DPH at 28 °C for 30 min, followed by washing with PBS, 7.5 μg/mL of AMB as a positive control. The fluorescence intensity was measured by a multifunctional fluorescent microplate reader at lex 350 nm and lem 425 nm. The experiment was conducted in triplicate.

### 4.8. Detection of Active Oxygen

The intracellular ROS content of *Candida albicans* was determined by using the DCFH-DA fluorescence method [45]. The concentration of *Candida albicans* was adjusted to 1–5 × 10^6^ CFU/mL, and then 2 × MIC Cecropin was used to incubate at 28 °C for different time intervals. After that, DCFH-DA with a final concentration of 2 μm was added and incubated in the dark for 30 min; AMB was used as the positive control (7.5 μg/mL). The fluorescence intensity was measured by a multifunctional fluorescent microplate reader at lex 488 nm and lem 525 nm. At the same time, in order to test the effects of two antioxidants, vitamin C (VC) and glutathione (GSH), on ROS accumulation induced by Cecropin, the *Candida albicans* cells used in the experiment needed to be preincubated in the culture environment of 20 mM VC and GSH for 2 h in advance, and the subsequent experimental steps were as described above. The experiment was conducted in triplicate.

### 4.9. Mitochondrial Membrane Potential

The assessment of mitochondrial membrane potential was performed by using the fluorescent probe rhodamine 123 [46]. Briefly, the *Candida albicans* was inoculated in YPD medium and oscillated at 28 °C for culture to the logarithmic phase, and then the concentration was adjusted to 1 × 10^6^–5 × 10^6^ CFU/mL. After that, it was incubated with Cecropin at a concentration of 1.8 μg/mL for different time points (0 min, 40 min, 60 min, 120 min, and AMB-120 min); it was centrifuged to remove the precipitate and washed twice with phosphate buffer solution (pH = 7.2–7.4) before it was re-suspended into the bacterial suspension at the same concentration. Each sample was added with a final concentration of 10 μm Rh-123 solution, which was incubated at 28 °C for 30 min under dark conditions and washed with PBS 2 or 3 times. Samples of 20 μL were viewed under an inverted fluorescence microscope in a blue light area, the fluorescence intensity (488 nm excitation light and 530 nm absorption light) was measured by using a multifunctional fluorescent microplate reader, and the changes of mitochondrial membrane potential were judged based on the fluorescence intensity. The experiment was conducted in triplicate.

## 5. Conclusions

Thus, Cecropin had high antifungal activity against *Candida albicans*, and it could simultaneously destroy the cell wall and cell membrane and cause mitochondrial dysfunction, thereby exerting antifungal activity. Given the growing problem of resistance in traditional therapies, as well as the high activity of antibacterial peptides and their difficulty in developing resistance, it is possible to open up an alternative therapy for the key task of developing new antifungal drugs.

## Data Availability

Not applicable.
